# Urine Oxidative Stress Biomarkers as Novel Biomarkers in Interstitial Cystitis/Bladder Pain Syndrome

**DOI:** 10.3390/biomedicines10071701

**Published:** 2022-07-14

**Authors:** Yuan-Hong Jiang, Jia-Fong Jhang, Han-Chen Ho, Dan-Yun Chiou, Hann-Chorng Kuo

**Affiliations:** 1 Department of Urology, Hualien Tzu Chi Hospital, Buddhist Tzu Chi Medical Foundation, Hualien 970, Taiwan; redeemerhd@gmail.com (Y.-H.J.); alur1984@tzuchi.com.tw (J.-F.J.); ytc9551@gmail.com (D.-Y.C.); 2 Department of Urology, School of Medicine, Tzu Chi University, Hualien 970, Taiwan; 3 Department of Anatomy, Tzu Chi University, Hualien 970, Taiwan; hcho@gms.tcu.edu.tw

**Keywords:** oxidative stress, hypoxia, interstitial cystitis, bladder pain syndrome, urine biomarker

## Abstract

Both hypoxia and chronic suburothelial inflammation are important pathophysiological findings in patients with interstitial cystitis/bladder pain syndrome (IC/BPS). This study investigated the roles of urine oxidative stress biomarkers and inflammatory cytokines in patients with IC/BPS. Urine samples were collected from 159 IC/BPS patients and 28 controls. The targeted analytes included oxidative stress biomarkers (8-OHdG, 8-isoprostane, and total antioxidant capacity) and inflammatory cytokines (MCP-1, RANTES, CXCL10, Eotaxin, MIP-1β, and IL-8). IC/BPS patients were classified into four clinical subgroups, based on the glomerulation grade and the maximal bladder capacity under anesthesia. Patients with IC/BPS had urine oxidative stress biomarkers and inflammatory cytokines profiles that were distinct from those of the controls and among each subgroup. Both 8-OHdG and 8-isoprostane showed a high diagnostic ability to distinguish type 2 IC/BPS patients (as classified by the European Society for the Study of Interstitial Cystitis) from controls. Additionally, they both showed positive and negative correlations with the glomerulation grade and the maximal bladder capacity under anesthesia, respectively. Limitations included intra-individual variation and sex influence. Urine oxidative stress biomarkers might have a role in diagnosing IC/BPS and differentiating its clinical subtypes. In addition to inflammatory cytokines, urine oxidative stress biomarkers have the potential to be novel biomarkers in patients with IC/BPS.

## 1. Introduction

Interstitial cystitis/bladder pain syndrome (IC/BPS) is a chronic inflammatory disorder of the urinary bladder, characterized by bladder pain, that is associated with urinary frequency, nocturia, and sterile urine [1]. While the etiology of IC/BPS remains unclear, it is considered multifactorial, with complex pathophysiology [2,3]. Defective and damaged bladder urothelium, mast cell activation, neurogenic inflammation, occult infection, autoimmunity and allergy, and hypoxia have been proposed as pathomechanisms [1,2]. Urothelial dysfunction and suburothelial inflammation are important pathologic findings in patients with IC/BPS [4]; their urine inflammatory protein profiles are reportedly distinct from those of controls [5] or patients with an overactive bladder [6]. With the increased understanding of IC/BPS pathophysiology, the development of IC/BPS biomarkers has received more attention.

Previous studies have proven that patients with IC/BPS have significantly decreased bladder perfusion during filling and at capacity [7,8], increased expression of hypoxia-inducible factor (HIF)-1α and vascular endothelial growth factor, and immature vascularization of the bladder tissue [9,10]. Bladder tissue hypoxia was thought to play an important role in the pathogenesis of IC/BPS and to be a potential therapeutic marker [11].

Both hypoxia and inflammation-inducing stimuli could generate reactive oxygen species, which serve as mediators in both physiological and pathological signaling transductions [12]. The excessive production of reactive oxygen species can result in oxidative stress, which may damage cellular DNA, lipids, and proteins, thereby influencing the structure and function of the target tissue, with a pathological link to many human diseases [13,14,15]. The application of oxidative stress (such as 8-hydroxy-2-deoxyguanosine (8-OHdG) and F2-isoprostane) and antioxidant biomarkers (such as total antioxidant capacity (TAC)) have been reported in some animal studies of bladder outlet obstruction (BOO) [16], but few human studies have been conducted, and none have considered IC/BPS.

For IC/BPS, both hypoxia and chronic suburothelial inflammation are important pathologic findings; however, to the best of our knowledge, no study has investigated the oxidative stress state in urine. Therefore, this study was conducted to investigate the roles and diagnostic values of urine oxidative stress biomarkers, as well as inflammatory cytokines, in patients with IC/BPS.

## 2. Materials and Methods

### 2.1. Patients

From February 2014 to December 2020, we enrolled 159 consecutive clinical IC/BPS patients from the department of urology at a single medical center (Hualien Tzu Chi Hospital, Taiwan). This study was approved by the Institutional Review Board and Ethics Committee of Buddhist Tzu Chi General Hospital (Nos. IRB105-31-A and IRB107-175-A). All study patients and controls were informed of the rationale and procedures of this study and signed an informed consent form.

The diagnostic criteria for IC/BPS, based on the proposed guidelines of European Society for the Study of Interstitial Cystitis (ESSIC), constituted “chronic pelvic pain, pressure, or discomfort perceived to be related to the urinary bladder, accompanied by at least one other urinary symptom, such as a persistent urge to void or urinary frequency, for more than six months”, with the exclusion of similar diseases [17]. Study patients received cystoscopy with hydrodistention under general anesthesia and were classified according to ESSIC as type 1 or 2 (without or with glomerulations, respectively). We excluded patients with Hunner’s lesions (ESSIC type 3). A total of 28 women with genuine stress urinary incontinence and without other storage or voiding dysfunction were invited as controls. Detailed inclusion and exclusion criteria were identical to those in our previous study [5].

### 2.2. Clinical Investigation

In enrolled IC/BPS patients, the assessment of clinical symptoms included the O’Leary–Saint symptom score, interstitial cystitis symptom index, interstitial cystitis problem index, and visual analog pain scale score. The grade of glomerulation and maximal bladder capacity under anesthesia (MBC) on cystoscopic hydrodistention were recorded. The patients were then classified into four clinical subtypes, based on the grade of glomerulation and MBC [18], including: (1) patients with glomerulation grade ≤ 1 and MBC ≥ 760 mL; (2) patients with glomerulation grade ≤ 1 and MBC < 760 mL; (3) patients with glomerulation grade ≥ 2 and MBC ≥ 760 mL; and (4) patients with glomerulation grade ≥ 2 and MBC < 760 mL.

### 2.3. Urine Biomarkers Investigation

Urine samples were collected from all study patients and controls before cystoscopic hydrodistention and surgery, respectively. Urine was self-voided when the patients had a full bladder sensation. Urinalysis was performed simultaneously to confirm an infection-free status before the urine samples were stored. A total of 50 mL of urine was put on ice immediately and transferred to the laboratory for preparation. The samples were centrifuged at 1800 rpm for 10 min at 4 °C. The supernatant is separated into aliquots in 1.5 mL tubes (1 mL per tube) and preserved in a freezer at −80 °C. Before further analyses were performed, the frozen urine samples were centrifuged at 12,000 rpm for 15 min at 4 °C, and the supernatants were set aside for subsequent measurements.

### 2.4. Quantification of 8-OHdG, 8-Isoprostane, and TAC

The quantification of 8-OHdG, 8-isoprostane, and TAC in urine samples was performed in accordance with the respective manufacturer’s instructions (8-OHdG ELISA kit, Biovision, Waltham, MA, USA; 8-isoprostane ELIZA kit, Enzo, Farmingdale, NY, USA; Total Antioxidant Capacity Assay Kit, abcam, Cambridge, MA, USA). The laboratory procedures were similar to those reported in a previous study [19].

### 2.5. Quantification of Inflammatory Cytokines

Inflammation-related cytokines in the urine samples were assayed using commercially available microspheres with the Milliplex^®^ human cytokine/chemokine magnetic bead-based panel kit (Millipore, Darmstadt, Germany). Six targeted analytes, including Eotaxin, interleukin-8 (IL-8), chemokine (C-X-C motif) ligand 10 (CXCL10), macrophage chemoattractant protein-1 (MCP-1), macrophage inflammatory protein 1β (MIP-1β), and ‘regulated upon activation, normal T cell expressed and presumably secreted’ (RANTES) values were measured with the multiplex kit, catalog number HCYTMAG-60K-PX30. The laboratory procedures used to quantify these targeted analytes were similar to those reported in previous studies [5,6].

### 2.6. Statistical Analysis

Continuous variables are represented as means ± standard deviations, and categorical data are represented as numbers and percentages. For each targeted analyte, the values outside the range between the means ± 3 of standard deviations in either the study or the control group were defined as outliers and were excluded from further analysis. The clinical data and the urine samples’ targeted analytes levels between the study and control groups and among the study subgroups and controls were analyzed using an analysis of variance.

Receiver operating characteristics curves were generated to assess the ability of each targeted analyte to distinguish ESSIC type 2 IC/BPS patients from individuals in the control group, and the areas under the receiver operating characteristic curves (AUC) were calculated. Multivariate logistic regression models were constructed to control confounding factors for each targeted analyte, and the odds ratio (OR) was calculated. Linear regression analysis with Pearson correlation was carried out to determine the relationship between clinical characteristics and the urine samples’ targeted analyte levels. All calculations were performed using SPSS Statistics for Windows, Version 25.0 (IBM Corp., Armonk, NY, USA). Differences were considered statistically significant for *p*-values < 0.05.

## 3. Results

Eligible IC/BPS patients included 139 women and 20 men, with a mean age of 54.4 ± 12.7 years (range: 21–88 years), which was similar to that of the 28 controls (mean age: 58.6 ± 9.9 years, range: 39–75 years) (Table 1). Based on the findings regarding cystoscopic hydrodistention, 42 (26.4%) and 117 (73.6%) of the IC/BPS patients were classified as ESSIC types 1 and 2, respectively. The clinical characteristics and symptom scores were similar among the patient types, except that ESSIC type 2 IC/BPS patients had a lower body mass index and a lower MBC.

Table 2 reveals the urine samples’ targeted analyte levels between IC/BPS patients and controls. For each targeted analyte, the numbers of outliers within the IC/BPS and control groups ranged from 0 to 6 and from 0 to 1, respectively, and all were less than 4%. The urine oxidative stress biomarkers (including elevated 8-OHdG and 8-isoprostane levels) and inflammatory cytokines profiles (including elevated MCP-1, RANTES, CXCL10, and Eotaxin levels) of the IC/BPS patients were distinct from that of the controls. Additionally, urine 8-OHdG levels were significantly higher in ESSIC type 2 IC/BPS patients than those in ESSIC type 1 patients.

**Figure 1:**
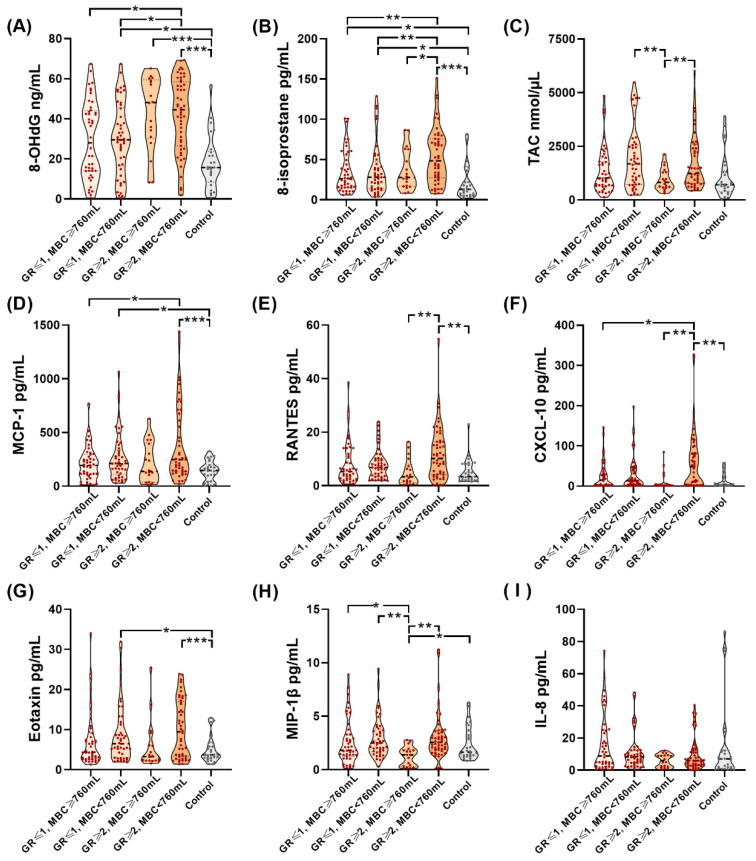
Violin plots of levels of urine biomarkers among different subgroups of patients with IC/BPS and control patients. Urine levels of (**A**) 8-OhdG, (**B**) 8-isoprostane, (**C**) TAC, (**D**) MCP-1, (**E**) RANTES, (**F**) CXCL-10, (**G**) Eotaxin, and (**H**) MIP-1β, but not (**I**) IL-8 were significantly different among different subgroups of patients with IC/BPS and control patients. *: *p*-value < 0.05, **: *p*-value < 0.01, ***: *p*-value < 0.001.

Table 3 and Figure 1 reveal the urine samples’ targeted analyte levels among the different subgroups of IC/BPS patients and controls. Among the subgroups of IC/BPS patients and the control group, there were significant differences in the expression of urine oxidative stress biomarkers and inflammatory cytokines. IC/BPS patients with a glomerulation grade ≥ 2 and MBC < 760 mL had the highest urine 8-OHdG and 8-isoprostane levels among subgroups.

Table 4 summarizes the diagnostic values of each urine analyte, to distinguish ESSIC type 2 IC/BPS patients from controls. Urine oxidative stress biomarkers, such as 8-OHdG and 8-isoprostane, demonstrated a high AUC (i.e., >0.7), acceptable sensitivity (75.2% and 68.4%, respectively) and specificity (80.8% and 76.9%, respectively). Additionally, urine 8-OHdG and 8-isoprostane had higher AUC levels than the other inflammatory cytokines.

Multivariate logistic regression models, adjusting for age, sex, body mass index, and the comorbidity of diabetes mellitus, revealed the OR of the diagnostic value of targeted analytes (Table 5). The urine samples’ targeted analytes that differentiated overall IC/BPS patients from controls included MCP-1 (OR 2.030), 8-OHdG (OR 1.687), 8-isoprostane (OR 1.557), Eotaxin (OR 1.141), and RANTES (1.012). More importantly, 8-OHdG was the only independent urine analyte to differentiate ESSIC type 2 from ESSIC type 1 IC/BPS patients.

Table 6 demonstrates the correlation coefficients between the clinical characteristics of ESSIC type 2 patients and urine biomarkers levels. Both urine 8-OHDdG and 8-isoprostane levels were positively correlated with the glomerulation grade and negatively correlated with MBC. All targeted oxidative stress biomarkers and inflammatory cytokines were negatively correlated with the MBC, except in the case of IL-8. However, in ESSIC type 1 patients, there were no significant correlations between their clinical characteristics and the urine samples’ targeted analyte levels.

## 4. Discussion

To the best of our knowledge, this is the first clinical study to investigate the roles of oxidative stress biomarkers in patients with IC/BPS. We found that patients with IC/BPS had distinct urine oxidative stress biomarkers and inflammatory cytokine profiles compared to those of the control groups. The urine analytes, 8-OHdG and 8-isoprostane, were independently capable of discriminating ESSIC type 2 IC/BPS patients from controls, showing an even higher AUC than selected inflammatory cytokines. Additionally, we noted significant correlations between the clinical characteristics of ESSIC type 2 IC/BPS patients and urine oxidative stress biomarkers. The non-invasive and convenient approach of urine oxidative stress biomarker analysis could provide important clinical information in the diagnosis and mapping of the clinical characteristics of IC/BPS, suggesting their potential for serving as novel biomarkers in patients with IC/BPS.

Considering that 8-OHdG is a stable end-product of DNA oxidation, the levels are not affected by long-term storage of the urine specimen, even at −20 °C [20]. F2-isoprostane is formed by the free radical-induced peroxidation of arachidonic acid [21]; it is a sensitive but chemically stable compound, not affected by diet, and detectable in all normal tissue and biological fluids (including urine) [21]. TAC reflects the cumulative effect of all antioxidants from various endogenous anti-oxidative defense systems against the harmful activities caused by oxidative stress [22]. Tissue hypoxia and the hypoxia-induced signaling pathways play critical roles in the disease progression and bladder remodeling of BOO [23,24]. One recent review disclosed that 8-OHdG, F2-isoprostane, and TAC were utilized as oxidative stress and antioxidant biomarkers in BOO-related urinary dysfunction [16]. Alongside the understanding of hypoxia-related pathophysiology in IC/BPS, these biomarkers might also be applied in IC/BPS.

Chronic bladder ischemia and hypoxia might produce oxidative stress, which leads to further bladder denervation and tissue damage; HIF plays an important role in the related signaling pathway [25]. Bladder tissue hypoxia [7,8] and increased expressions of HIF-1α [9] were confirmed in IC/BPS patients. However, no further investigations regarding the oxidative stress state in either the tissue or urine specimens have been performed. This study revealed that urine 8-OHdG and 8-isoprostane levels were significantly elevated in IC/BPS patients and correlated with clinical characteristics, including positive and negative correlations with glomerulation grade and MBC, respectively. The results suggested that these urine oxidative stress biomarkers might not only reflect the underlying hypoxia-related pathology but also the consequent bladder function (such as the glomerulation grade and MBC) after oxidative damage from chronic hypoxia in IC/BPS patients.

MCP-1, RANTES, and CXCL10 are chemokines involved in peripheral neurological inflammation responses [26]. Eotaxin is implicated in many pathologic conditions caused by eosinophilic inflammation [27]. These inflammatory chemokines and cytokines were reported to be elevated in IC/BPS urine specimens and are considered to be useful biomarkers in IC/BPS patients [5]. The higher AUC of 8-OHdG and 8-isoprostane for discriminating ESSIC type 2 IC/BPS patients from control patients than those of these selected inflammatory chemokines and cytokines might be attributed to certain characteristics of 8-OHdG and 8-isoprostane, such as their increased stability in urine and decreased intra-individual variation, confounded by the other pro-inflammatory conditions. The urine 8-OHdG level might be a good indicator of bladder oxidative stress state in patients with IC/BPS. In this study, the urine 8-OHdG level was not only significantly elevated in overall IC/BPS patients but it was also the only independent urine analyte to differentiate ESSIC type 2 from ESSIC type 1 ICBPS patients.

IC/BPS as a disease has the characteristics of a multifactorial etiopathophysiology [1,2] and heterogeneous clinical phenotyping [28]. In this study, the urine 8-OHdG levels in ESSIC type 2 IC/BPS patients were significantly greater than those in ESSIC type 1 patients. However, the correlation between the urine samples’ targeted analyte levels (both oxidative stress biomarker and inflammatory cytokines) and the clinical characteristics were significant in only ESSIC type 2 and not in ESSIC type 1 IC/BPS patients. This suggests that the pathophysiological roles of bladder hypoxia and specific neurologic or eosinophilic inflammation were less substantial in ESSIC type 1 IC/BPS patients than in ESSIC type 2 IC/BPS patients. The different exhibited protein profiles and biochemical contents might reflect the distinct pathophysiologies and intrinsic bladder conditions. The application of a cluster of biomarkers might elevate the diagnostic values and may prove to be a better strategy for the development of IC biomarkers. Additionally, IC/BPS patients with different MBC and glomerulation grades were reported to have different treatment outcomes [18]. In this study, there were significant differences in the expressions of the urine oxidative stress biomarkers and inflammatory cytokines among the subgroups of IC/BPS patients and controls. These biomarkers might have potential prognostic roles, which must be validated in future studies.

There were several limitations to this study. First, most of the enrolled study patients and controls were women, and there might be differences in response between the sexes. Second, all controls were patients with genuine stress urinary incontinence and were not drawn from the healthy general population, even though this study design was consistent with previous studies [5,6]. Third, although oxidative stress biomarkers are more stable compounds than inflammatory cytokines, there might be intra-individual variations. Fourth, the overall hypoxia and oxidative stress status within the bladder were not totally contributed by the bladder pathology caused by IC/BPS. Both systemic inflammatory diseases and comorbidities and local bladder insults might affect the expressions of urine biomarkers. Finally, the expression levels of some biomarkers in the urine samples exhibited extreme values. Although the percentage of outliers was low, we excluded them from our analysis.

## 5. Conclusions

Patients with IC/BPS had urine oxidative stress biomarkers and inflammatory cytokine profiles that were distinct from those of the control group. Both urine 8-OHdG and 8-isoprostane levels, being indicators of oxidative stress, demonstrated a high ability to discriminate IC/BPS patients from control patients, and correlated with the pathologic conditions of the bladder. In addition to inflammatory cytokines, urine oxidative stress biomarkers have the potential to be novel biomarkers in patients with IC/BPS.

## Figures and Tables

**Table 1:** Clinical characteristics of patients with IC/BPS and the control patients.

	IC/BPS					
	ESSIC Type 1 (N = 42, 26.4%)	ESSIC Type 2 (N = 117, 73.6%)	Overall (N = 159, 100%)	Control (N = 28)	*p*-Value *	*p*-Value ^#^
Age	56.9 ± 10.9 (28–78)	53.5 ± 13.2 (21–88)	54.4 ± 12.7 (21–88)	58.6 ± 9.9 (39–75)	0.145	0.101
Sex	F40, M2	F99, M18	F139, M20	F 28	0.075	0.048
DM	8 (19.0%)	10 (8.5%)	18 (11.3%)	5 (17.9%)	0.087	0.350
BMI	25.88 ± 4.24	23.00 ± 4.07	23.76 ± 4.30	25.66 ± 4.03	<0.001	0.031
VAS	4.4 ± 2.6	4.5 ± 2.8	4.5 ± 2.7		0.766	
ICSI	9.8 ± 4.7	10.9 ± 4.3	10.6 ± 4.4		0.194	
ICPI	10.4 ± 4.5	10.7 ± 3.7	10.6 ± 3.9		0.625	
OSS	20.2 ± 8.8	21.7 ± 7.5	21.3 ± 7.8		0.272	
MBC (mL)	782.1 ± 192.5	711.1 ± 176.8	729.9 ± 183.2		0.031	

Note: DM, diabetes mellitus; BMI, body mass index; VAS, visual analog scale; ICSI, interstitial cystitis symptom index; ICPI, interstitial cystitis problem index; OSS, O’Leary-Saint score; MBC, maximal bladder capacity under anesthesia. *: *p*-values between ESSIC type 1 and type 2 IC/BPS patients. ^#^: *p*-values between overall IC/BPS patients and controls.

**Table 2:** Levels of urine biomarkers between IC/BPS and control groups.

	IC/BPS					
Urine Biomarkers ^@^	(A) ESSIC Type 1 N = 42	(B) ESSIC Type 2 N = 117	Overall N = 159	(C) Control N = 28	*p*-Value ^#^	*p*-Value ^$^
8-OHdG	25.81 ± 18.44 (0)	38.67 ± 18.68 * (0)	35.27 ± 19.41 (0)	18.33 ± 13.48 (0)	<0.001	<0.001
8-isoprostane	36.47 ± 26.4 * (0)	44.32 ± 33.11 * (3)	42.20 ± 31.56 (3)	20.69 ± 21.15 (0)	0.169	<0.001
TAC	1641.8 ± 1317.2 (0)	1597.4.2 ± 1226.0 (6)	1610.6 ± 1247.5 (6)	1119.4 ± 1064.1 (0)	0.845	0.060
MCP-1	237.57 ± 211.06 * (2)	297.47 ± 276.06 * (3)	281.91 ± 261.41 (5)	142.25 ± 93.02 (1)	0.214	<0.001
RANTES	7.97 ± 8.00 (0)	9.34 ± 8.21 * (1)	8.97 ± 8.15 (1)	5.34 ± 4.56 (1)	0.354	0.001
CXCL 10	25.76 ± 38.68 (1)	41.04 ± 57.59 * (1)	37.05 ± 53.61 (2)	14.76 ± 18.63 (1)	0.117	<0.001
Eotaxin	8.25 ± 8.03 (1)	8.52 ± 6.8 * (2)	8.45 ± 7.12 (3)	4.88 ± 3.21 (1)	0.836	<0.001
MIP-1β	2.82 ± 2.24 (1)	2.65 ± 1.96 (2)	2.69 ± 2.03 (3)	2.44 ± 1.59 (0)	0.635	0.533
IL-8	11.71 ± 13.46 (1)	11.45 ± 13.12 (2)	11.52 ± 13.17 (3)	14.62 ± 24.13 (1)	0.914	0.520

Note: 8-OHdG, 8-hydroxy-2-deoxyguanosine; TAC, total antioxidant capacity; MCP-1, macrophage chemoattractant protein-1; RANTES, regulated upon activation, normal T cell expressed and presumably secreted; CXCL10, chemokine (C-X-C motif) ligand 10; MIP-1β, macrophage inflammatory protein 1β. (): indicates the number of outliers. *: *p*-values < 0.05 when compared with controls. ^#^: *p*-values between ESSIC type 1 and type 2 IC/BPS patients. ^$^: *p*-values between overall IC/BPS patients and controls. ^@^: units: all pg/mL, except ng/mL in 8-OHdG, and mmol/μL in TAC.

**Table 3:** Levels of urine biomarkers among different subgroups of patients with IC/BPS and the control patients.

	IC/ BPS						
Urine Biomarkers ^@^	(A) GR ≤ 1, MBC ≥ 760 mL N = 44	(B) GR ≤ 1, MBC < 760 mL N = 46	(C) GR ≥ 2, MBC ≥ 760 mL N = 18	(D) GR ≥ 2, MBC < 760 mL N = 51	Control N = 28	*p*-Value *	Post Hoc Analysis
8-OHdG	29.56 ± 19.31 (0)	30.12 ± 18.05 (0)	42.97 ± 18.39 (0)	42.13 ± 18.40 (0)	18.33 ± 13.48 (0)	<0.001	A, B, vs. D B, C, D vs. E
8-isoprostane	35.21 ± 26.55 (0)	37.61 ± 32.18 (1)	37.28 ± 25.57 (0)	54.51 ± 34.28 (2)	20.69 ± 21.15 (0)	<0.001	A, B, C vs. D A, B, D vs. E
TAC	1399.5 ± 1080.4 (0)	1926.6 ± 1431.2 (1)	962.8 ± 514.1 (0)	1753.4 ± 1306.1 (5)	1609.6 ± 1247.5 (6)	0.003	B vs. C C vs. D
MCP-1	209.3 ± 156.9 (1)	263.7 ± 215.8(1)	218.2 ± 186.4 (0)	387.92 ± 355.17 (3)	142.3 ± 93.02 (1)	<0.001	A vs. D B, D vs. E
RANTES	7.98 ± 8.02 (0)	8.09 ± 5.67 (1)	5.09 ± 5.02 (0)	11.98 ± 10.01 (0)	5.34 ± 4.56 (1)	<0.001	C, E vs. D
CXCL 10	26.01 ± 34.24 (0)	31.41 ± 43.18 (2)	12.55 ± 22.84 (2)	60.09 ± 72.69 (0)	14.76 ± 18.63 (1)	<0.001	A, C, E vs. D
Eotaxin	7.30 ± 6.93 (0)	8.73 ± 7.30 (1)	5.85 ± 6.15 (1)	10.20 ± 7.16 (2)	4.88 ± 3.21 (1)	0.004	B, D vs. E
MIP-1β	2.63 ± 2.16 (1)	2.97 ± 1.87 (1)	1.27 ± 0.91 (2)	3.01 ± 2.17 (0)	2.44 ± 1.59 (0)	0.016	A, B, D, E vs. C
IL-8	16.3 ± 18.24 (0)	11.10 ± 11.13 (3)	5.99 ± 3.96 (3)	9.69 ± 10.37 (0)	14.62 ± 24.13 (1)	0.090	

Note: 8-OHdG, 8-hydroxy-2-deoxyguanosine; TAC, total antioxidant capacity; MCP-1, macrophage chemoattractant protein-1; RANTES, regulated upon activation, normal T cell expressed and presumably secreted; CXCL10, chemokine (C-X-C motif) ligand 10; MIP-1β, macrophage inflammatory protein 1β; GR, grade of glomerulation; MBC, maximal bladder capacity under anesthesia. (): indicates the number of outliers. *: *p*-values among different subgroups of IC/ BPS patients and control patients. ^@^: units: all pg/mL, except ng/mL in 8-OhdG, and mmol/μL in TAC.

**Table 4:** Diagnostic values of urine biomarkers in patients with ESSIC type 2 IC/BPS (from controls).

Urine Biomarkers	AUC	Cut-off Value ^@^:	Sensitivity (%)	Specificity (%)	PPV (%)	NPV (%)
8-OHdG	0.799	24.970	75.2%	80.8%	94.6%	42.0%
8-isoprostane	0.755	22.245	68.4%	76.9%	92.9%	35.7%
MCP-1	0.681	199.070	54.4%	77.8%	91.2%	28.8%
RANTES	0.655	8.770	41.4%	88.9%	94.1%	26.1%
TAC	0.649	845.210	64.9%	65.4%	88.9%	30.4%
Eotaxin	0.645	6.950	47.8%	85.2%	93.2%	27.7%
CXCL10	0.629	58.425	26.7%	100.0%	100.0%	24.1%
IL 8	0.556	2.165	84.4%	37.0%	85.1%	35.7%
MIP-1β	0.555	1.570	73.0%	50.0%	85.7%	31.1%

Note: 8-OHdG, 8-hydroxy-2-deoxyguanosine; TAC, total antioxidant capacity; MCP-1, macrophage chemoattractant protein-1; RANTES, regulated upon activation, normal T cell expressed and presumably secreted; CXCL10, chemokine (C-X-C motif) ligand 10; MIP-1β, macrophage inflammatory protein 1β; AUC, area under the curve of the receiver operating characteristic; PPV, positive predictive value; NPV, negative predictive value. ^@^: units: all pg/mL, except ng/mL in 8-OHdG, and mmol/μL in TAC.

**Table 5:** Multivariate models (adjusting for age, gender, BMI, and DM), revealing the diagnostic values of targeted urine biomarkers.

	*p*-Value	Odds Ratio	95% CI	Odds Ratio Units *
IC/BPS (Total) vs. control				
MCP-1	0.002	2.030	1.286–3.205	100
8-OHdG	<0.001	1.687	1.258–2.264	10
8-isoprostane	0.002	1.557	1.176–2.060	10
Eotaxin	0.017	1.141	1.024–1.271	1
RANTES	0.048	1.102	1.001–1.213	1
IC/BPS (ESSIC type 2) vs. control				
MCP-1	0.002	2.362	1.377–4.050	100
8-OHdG	0.000	2.056	1.448–2.919	10
8-isoprostane	0.003	1.512	1.152–1.987	10
CXCL 10	0.030	1.224	1.020–1.468	10
Eotaxin	0.014	1.165	1.032–1.316	1
RANTES	0.036	1.116	1.007–1.238	1
IC/BPS (ESSIC type 2) vs. IC/BPS (ESSIC type 1)				
8-OHDG	0.001	1.456	1.161–1.826	10

*: units: all pg/mL, except ng/mL in 8-OHdG, and mmol/μL in TAC.

**Table 6:** The correlation coefficient (r-value) between urine biomarker levels and the clinical characteristics in patients with ESSIC type 2 IC/BPS.

Urine Cytokines *	Grade of Glomerulation	MBC	VAS	ICSI	ICPI	OSS
8-OHdG	0.217	−0.234	n.s.	n.s.	n.s.	n.s.
8-isoprostane	0.190	−0.237	n.s.	n.s.	n.s.	n.s.
TAC	n.s.	−0.275	n.s.	−0.276	n.s.	−0.206
MCP-1	0.205	−0.268	n.s.	n.s.	n.s.	n.s.
RANTES	n.s.	−0.344	n.s.	n.s.	n.s.	n.s.
CXCL 10	n.s.	−0.305	n.s.	n.s.	n.s.	n.s.
Eotaxin	n.s.	−0.350	n.s.	n.s.	n.s.	n.s.
MIP-1β	n.s.	−0.249	n.s.	n.s.	n.s.	n.s.
IL-8	−0.207	n.s.	n.s.	n.s.	n.s.	n.s.

Note: MBC, maximal bladder capacity under anesthesia; VAS, visual analog scale; ICSI, interstitial cystitis symptom index; ICPI, interstitial cystitis problem index; OSS, O’Leary- Saint score; n.s., not significant; 8-OHdG, 8-hydroxy-2-deoxyguanosine; TAC, total antioxidant capacity; MCP-1, macrophage chemoattractant protein-1; RANTES, regulated upon activation, normal T cell expressed and presumably secreted; CXCL10, chemokine (C-X-C motif) ligand 10; MIP-1β, macrophage inflammatory protein 1β. *: units: all pg/mL, except ng/mL in 8-OHdG, and mmol/μL in TAC.
